# Clonal dissemination of carbapenem-resistant ST477 *Klebsiella michiganensis* co-producing NDM-1, SFO-1, and VEB-3 in a Chinese hospital

**DOI:** 10.3389/fcimb.2025.1679043

**Published:** 2025-10-22

**Authors:** Hao Liu, Chao Yan, Meiling Jiao, Juntian Jiang, Sibo Wang, Zhuo Wei, Fupin Hu, Xuesong Xu

**Affiliations:** ^1^ Department of Laboratory Medicine, China-Japan Union Hospital of Jilin University, Changchun, China; ^2^ Institute of Antibiotics, Huashan Hospital, Fudan University, Shanghai, China; ^3^ Joint Laboratory of Hospital & Enterprise for Pathogen Diagnosis of Drug-Resistant Bacterial Infections and Innovative Drug R&D, Shanghai, China; ^4^ Medical Laboratory Center, JiLin Provincial People’s Hospital, Changchun, China; ^5^ Key Laboratory of Clinical Pharmacology of Antibiotics, Ministry of Health, Shanghai, China

**Keywords:** *Klebsiella michiganensis*, carbapenem resistance, clonal dissemination, *bla*_NDM-1_, whole-genome sequencing

## Abstract

**Objectives:**

To investigate the antimicrobial resistance mechanisms and intra-hospital clonal dissemination of carbapenem-resistant ST477 *Klebsiella michiganensis*.

**Methods:**

Between 14 December 2019 and 23 August 2020, six *K. michiganensis* isolates producing NDM-type carbapenemases were recovered from Jilin Provincial People’s Hospital in China. Antimicrobial susceptibility was determined using the broth microdilution method. Whole-genome sequencing (WGS) was performed for all isolates. Sequence typing (ST), resistance genes, and plasmid types were identified using the PubMLST, ResFinder, and PlasmidFinder databases, respectively. Conjugation experiments were conducted to assess plasmid transferability. Additionally, 344 publicly available *K. michiganensis* genomes were retrieved and used to construct a phylogenetic tree based on core-genome single nucleotide polymorphisms (SNPs).

**Results:**

WGS revealed that all six isolates belonged to ST477 and harbored *bla*_NDM-1_, *bla*_SFO-1_, and *bla*_VEB-3_. The maximum pairwise difference among the six isolates was only 8 SNPs, indicating clonal transmission. Antimicrobial susceptibility testing showed high-level resistance to imipenem, meropenem, and ceftazidime-avibactam, while susceptibility was retained to amikacin, aztreonam-avibactam, eravacycline, tigecycline, and colistin. Conjugation assays confirmed that the *bla*_NDM-1_-carrying plasmid was self-transmissible. Clinical data showed that four of the six patients had a history of transfer to the intensive care unit (ICU). Phylogenetic analysis combined with resistance gene profiling based on publicly available genomes revealed that 50% (175/350) of *K. michiganensis* isolates from human hosts carried carbapenem resistance genes. Notably, Isolates from China exhibited a higher carriage rate of carbapenemase genes (76.1%, 51/67). No ST477-related genomes were identified in current public datasets.

**Conclusions:**

This study is the first to report the clonal dissemination of ST477 *K. michiganensis* harboring *bla*_NDM-1_ in a Chinese hospital.

## 1 Introduction

*Klebsiella oxytoca* is an opportunistic pathogen that can be detected in the feces of approximately 8–10% of healthy adults (Savino et al., 2009). With the advancement of genomics, researchers have discovered that *K. oxytoca* is not a single species, but rather a complex comprising at least six distinct species (Yang et al., 2022), including *Klebsiella grimontii*, *Klebsiella huaxiensis*, *Klebsiella michiganensis*, *Klebsiella oxytoca*, *Klebsiella pasteurii*, and *Klebsiella* sp*allanzanii*. Among them, *K. michiganensis* is a newly recognized species, initially isolated from a domestic toothbrush holder (Saha et al., 2013). *K. michiganensis* shares typical phenotypic characteristics of the *Klebsiella* genus, and phylogenomic analysis has shown that it is most closely related to *K. oxytoca* (Wyres et al., 2020). As a result, it is often misidentified as *K. oxytoca* in routine clinical diagnostics. Although clinical reports of *K. michiganensis* remain limited, genomic studies of the *K. oxytoca* complex have revealed that *K. michiganensis* represents a significant proportion of isolates within this group (Gómez et al., 2021; Wan et al., 2023), suggesting its pathogenic potential may be underestimated.

The greatest clinical threat posed by *K. michiganensis* lies in its multidrug resistance, particularly the emergence of carbapenem-resistant strains (Zhang et al., 2021; Luo et al., 2023). Carbapenems are often regarded as the last line of defense for treating severe infections (Li et al., 2023b). Once *K. michiganensis* acquires resistance to carbapenems, conventional antibiotic therapies often fail, substantially increasing the difficulty of clinical treatment and the mortality rate of affected patients. The production of carbapenemases is the primary mechanism of carbapenem resistance in bacteria. In particular, strains producing metallo-β-lactamases (MBLs) can hydrolyse almost all β-lactam antibiotics except aztreonam. Among MBLs, New Delhi metallo-β-lactamase (NDM) is the most prevalent. Since its first identification in India in 2008 (Yong et al., 2009), NDM genes have been detected in a wide range of pathogens, including members of the *Enterobacterales*, *Acinetobacter*, and *Pseudomonas* genera (Johnson and Woodford, 2013). In addition, the horizontal transfer of plasmids carrying resistance genes further facilitates the dissemination of resistance among bacteria (Frankel et al., 2023). Mobile genetic elements (MGEs), such as insertion sequences, integrons, and transposons, frequently found in bacterial genomes, contribute to the interspecies spread of resistance genes through horizontal gene transfer (Mann et al., 2022; Macesic et al., 2023). Previous studies have shown that *K. michiganensis* also harbors diverse MGEs and possesses the ability to acquire and disseminate resistance genes through transposition and recombination (Campos-Madueno et al., 2021; Luo et al., 2022).

In this study, we characterized the molecular features of six clinical ST477 *K. michiganensis* isolates carrying *bla*_NDM-1_ and performed a comparative genomic analysis with publicly available *K. michiganensi*s genomes from human hosts worldwide. A phylogenetic tree was constructed to investigate the evolutionary characteristics of this species.

## 2 Materials and methods

### 2.1 Bacterial isolates

Between 14 December 2019 and 23 August 2020, six non-duplicate pathogenic strains were isolated from clinical specimens of six hospitalized patients at Jilin Provincial People’s Hospital (only the first isolate per patient was included).Initial identification by matrix-assisted laser desorption/ionization time-of-flight mass spectrometry (MALDI-TOF MS) indicated that all isolates belonged to the *Klebsiella oxytoca* complex. Specifically, strains KO24–256 and KO24–259 were isolated from pus, KO24–260 from a throat swab, KO24–263 from whole blood, KO24–297 from pleural fluid, and KO24–298 from urine. Final species identification by whole-genome sequencing confirmed all isolates as *K. michiganensis*. Polymerase chain reaction (PCR) was performed to screen for carbapenemase genes, including *bla*_KPC_, *bla*_NDM_, *bla*_IMP_, *bla*_OXA-48_, and *bla*_VIM_. Quality control strains for antimicrobial susceptibility testing included *Escherichia coli* ATCC 25922 and *Pseudomonas aeruginosa* ATCC 27853.

### 2.2 Antimicrobial susceptibility testing

Minimum inhibitory concentrations (MICs) of antimicrobial agents were determined using the broth microdilution method according to the guidelines of the Clinical and Laboratory Standards Institute (CLSI). The antibiotics tested included imipenem, meropenem, meropenem-vaborbactam, aztreonam-avibactam, ceftazidime-avibactam, piperacillin-tazobactam, ceftazidime, ceftriaxone, cefepime, aztreonam, amikacin, ciprofloxacin, sitafloxacin, colistin, tigecycline, and eravacycline. Interpretation of MIC results was performed as follows: colistin and aztreonam-avibactam were interpreted according to the European Committee on Antimicrobial Susceptibility Testing (EUCAST) breakpoints (The European Committee on Antimicrobial Susceptibility Testing, 2024); tigecycline and eravacycline were interpreted based on the US Food and Drug Administration (FDA) breakpoints for Enterobacterales (U.S. Food and Drug Administration., 2023); breakpoints for the remaining agents were interpreted according to the CLSI 2024 guidelines (Clinical and Laboratory Standards Institute., 2024).

### 2.3 Whole-genome sequencing and bioinformatic analysis

Genomic DNA was extracted from all isolates using a commercial DNA extraction kit following the manufacturer’s instructions. WGS was performed on all six clinical isolates using the Illumina short-read sequencing platform (Illumina, San Diego, CA, USA). In addition, isolate KO24–256 underwent long-read sequencing using the Nanopore platform (Oxford Nanopore Technologies, Oxford, UK) for further analysis. Raw reads were quality filtered and trimmed using Sickle (https://github.com/najoshi/sickle), and hybrid assembly was carried out with SPAdes v3.12.0 to generate high-quality draft genomes. The assembled genomes were analyzed using BLAST against several databases: sequence typing was performed with PubMLST; plasmid replicon types were identified with PlasmidFinder and PMLST; and acquired antimicrobial resistance genes were identified with ResFinder. Single nucleotide polymorphism (SNP) distances among isolates were calculated using Snippy v4.6.0 (https://github.com/tseemann/snippy). For KO24-256, open reading frames (ORFs) on the resistance plasmid were predicted and annotated using RAST v2.0 (https://rast.nmpdr.org) and BLAST (https://blast.ncbi.nlm.nih.gov/Blast.cgi).

### 2.4 Conjugation assay

To assess the transferability of the resistance plasmid, a conjugation assay was performed using isolate KO24–256 as the donor strain and *Escherichia coli* J53 as the recipient strain. The experiment was conducted using a standard liquid mating method. Donor and recipient strains were mixed at a 1:1 ratio and co-cultured in LB broth pre-warmed to 37 °C.Transconjugants were selected on Mueller–Hinton (MH) agar plates containing dual antibiotics: ampicillin (100 mg/L) and sodium azide (100 mg/L). Positive colonies growing on the selective medium were identified using MALDI-TOF MS. The presence of the *bla*_NDM-1_ gene in transconjugants was confirmed by PCR followed by Sanger sequencing. Plasmid DNA was extracted from transconjugants using the Qiagen Plasmid Midi Kit (Qiagen, Hilden, Germany) and further validated by short-read sequencing on the Illumina platform (Illumina, San Diego, CA, USA).

### 2.5 Phylogenetic analysis

To further investigate the evolutionary relationship of clinical ST477 *K. michiganensis* isolates, we downloaded all available *K. michiganensis* genomes sequenced between 2012 and 2024 from the Pathosystems Resource Integration Center (PATRIC). After filtering out low-quality assemblies and non-human host isolates, a total of 344 genomes were retained for analysis. These were combined with the six isolates from this study to construct a core genome-based phylogenetic tree. In total, 350 K*. michiganensis* genomes were included, representing isolates from 31 countries worldwide. Pairwise single nucleotide polymorphism (SNP) distances between genomes were calculated using Snippy v4.6.0 under default parameters. The phylogenetic tree was visualized and refined using FastTree and ChiPlot (Xie et al., 2023).

## 3 Results

### 3.1 Patient characteristics and epidemiological investigation

**Figure 1:**
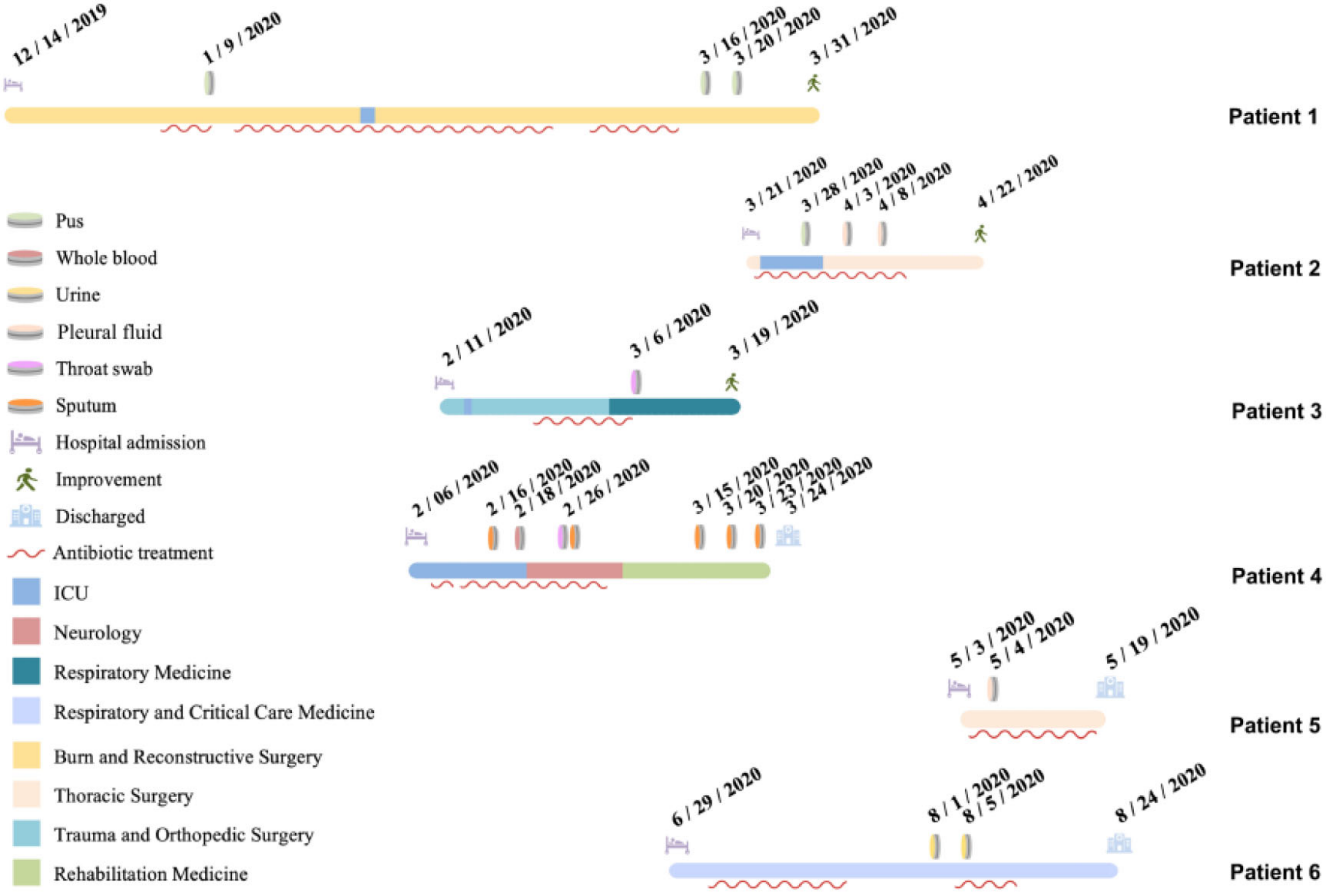
Epidemiological overview of the six patients included in this study. Each horizontal bar represents the hospitalization timeline of one patient(the timeline for Patient 6 is displayed separately), with different colors indicating different hospital wards. Red wavy lines denote periods of antibiotic treatment. Admission and discharge dates, as well as the dates of pathogen isolation (with colors indicating different specimen types), are marked numerically on the timeline. Different symbols at the end of each timeline indicate the discharge outcome of each patient.

The six hospitalized patients had a mean age of 63.5 years (range: 31–89 years). Five patients (all except Patient 4) underwent invasive procedures, including tracheal intubation, surgery, or puncture. The patients were admitted to five different hospital wards, and four (excluding Patient 5 and Patient 6) had a history of ICU admission. Notably, Patients 3 and 4 were hospitalized in the ICU during overlapping periods. Of particular interest, Patient 1 was transferred from a local hospital to the Department of Burns and Plastic Surgery at Jilin Provincial People’s Hospital on 14 December 2019 due to extensive burns. A pathogen was first isolated from wound pus on hospital day 27 (9 January 2020). Subsequently, the same organism was detected in the following specimens from other patients: sputum from Patient 4 (26 February 2020), throat swab from Patient 3 (6 March 2020), pus from Patient 2 (28 March 2020), and urine from Patient 5 (4 May 2020). The final isolate was obtained from the urine of Patient 6 on 5 August 2020. Among the six patients, three recovered and were discharged, while the other three were discharged after electing to discontinue treatment due to clinical deterioration. Detailed hospitalization histories and antibiotic usage for each patient are shown in (**Figure 1**).

### 3.2 Antimicrobial susceptibility and conjugation assays

Antimicrobial susceptibility testing showed that all six *K. michiganensis* isolates were resistant to imipenem (MIC range: 4 - 32 μg/mL), meropenem (MIC range: 8 - >32 μg/mL), and ceftazidime-avibactam (MIC>64 μg/mL). Isolate KO24–298 exhibited intermediate susceptibility to meropenem-vaborbactam, while the other five isolates were resistant. Among the antibiotics tested, only amikacin, aztreonam-avibactam, eravacycline, tigecycline, and colistin demonstrated good *in vitro* activity (**Table 1**).Conjugation assays confirmed that the *bla*_NDM-1_-carrying plasmid in KO24–256 could be successfully transferred to the recipient strain *E. coli* J53. The resulting transconjugant exhibited resistance to imipenem (MIC = 16 μg/mL), meropenem (MIC = 16 μg/mL), meropenem-vaborbactam (MIC=32 μg/mL), and ceftazidime-avibactam (MIC>64 μg/mL). Compared with the recipient strain, the MICs of imipenem, meropenem, meropenem-vaborbactam, and ceftazidime-avibactam in the transconjugant increased by at least 128-, 512-, 1024-, and 256-fold, respectively (**Table 1**).

**Table 1:** Antimicrobial susceptibility profiles of clinical isolates, the transconjugant, and the recipient strain.

Antibiotics	MIC (μg/mL)							
	*K. michiganensis*						E. coli J53 (NDM-1)	E. coli J53
	KO24-256	KO24-259	KO24-260	KO24-263	KO24-297	KO24-298		
Imipenem	16	32	16	16	16	4	16	0.125
Meropenem	32	>32	32	64	32	8	16	<=0.03
Meropenem-vaborbactam	64	64	32	64	64	8	32	<=0.03
Aztreonam-avibactam	1	1	1	0.5	0.25	1	0.25	0.06
Ceftazidime-avibactam	>64	>64	>64	>64	>64	>64	>64	0.25
Piperacillin-tazobactam	>256	>256	>256	>256	>256	>256	256	4
Ceftazidime	>32	>32	>32	>32	>32	>32	>32	0.5
Ceftriaxone	>32	>32	>32	>32	>32	>32	>32	<=0.25
Cefepime	>32	>32	>32	>32	>32	>32	>32	0.5
Aztreonam	>128	>128	>128	>128	128	>128	128	<=1
Amikacin	2	<=1	<=1	2	<=1	<=1	2	2
Ciprofloxacin	8	8	8	8	4	8	<=0.06	<=0.06
Sitafloxacin	1	1	1	1	1	1	<=0.06	<=0.06
Colistin	0.25	0.25	0.25	0.25	0.25	0.25	0.25	0.125
Tigecycline	0.5	0.5	0.25	0.25	0.25	0.25	0.25	0.125
Eravacycline	0.25	0.25	0.25	0.25	0.25	0.25	0.125	0.06

### 3.3 Whole-genome sequencing and plasmid characterization of *K. michiganensis* KO24-256

Whole-genome sequencing (WGS) analysis revealed that all six *K. michiganensis* isolates belonged to ST477 (allelic profile: *gapA 3*; *infB 41*; *mdh 20*; *pgi 21*; *phoE 20*; *rpoB 6*; *tonB 25*). All isolates harbored multiple resistance genes, including the carbapenemase gene (*bla*_NDM-1_), β-lactamase genes (*bla*_VEB-3_, *bla*_SFO-1_, *bla*_TEM-1B_, *bla*_OXY-1-1_), and genes conferring resistance to quinolones (*qnrS1*), sulfonamides (*sul1*, *sul2*), chloramphenicol (*floR*), tetracyclines (*tet(A)*), trimethoprim (*dfrA12*, *dfrA27*), macrolides (*mph(A)*), and rifampicin (*ARR-3*). Among the six isolates, aminoglycoside resistance genes varied slightly. Isolates KO24-256, KO24-260, KO24-263, and KO24–298 carried *aph(3’)-Ia*, *aph*(6)*-Id*, *aph(3’’)-Ib*, *aadA2*, and *aac(3)-IId*, whereas isolates KO24–259 and KO24–297 only carried *aph(3’)-Ia*, *aadA2*, and *aac(3)-IId*. Pairwise SNP analysis revealed a maximum of only 8 SNP differences among the six isolates, indicating clonal dissemination.

**Figure 2:**
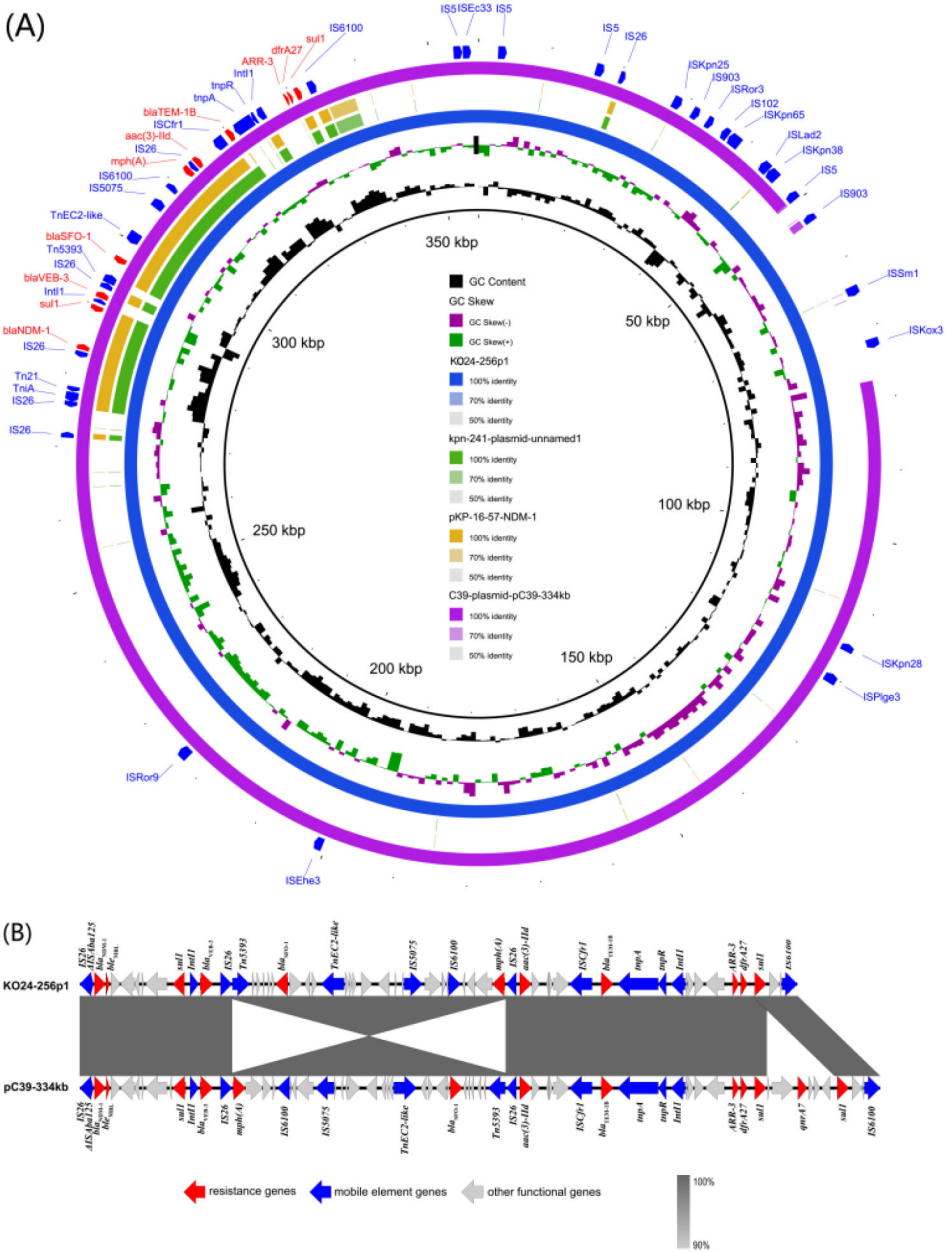
**(A)** Circular comparison of plasmid KO24-256p1 with plasmid pC39-334kb (CP061701), *K*. *pneumoniae* plasmid unnamed1 (CP053670), and pKP-16-57-NDM-1 (MZ836809). Different colored rings represent different plasmids. **(B)** Comparative genetic context of plasmid KO24-256p1 and pC39-334kb. Genes are indicated by arrows and colored based on their predicted functional categories.

To obtain a more complete genomic profile, long-read sequencing of *K. michiganensis* KO24–256 was performed using the Nanopore platform. The assembled genome had a total length of 6413616 bp, comprising a 5982250 bp circular chromosome and two circular plasmids, designated KO24-256p1 (356849 bp) and KO24-256p2 (74517 bp). The chromosome harbored the *aph(3’)-Ia* and *bla*_OXY-1–1_ genes, conferring resistance to aminoglycosides and β-lactams, respectively. Plasmid KO24-256p1 carried multiple resistance genes, including *bla*_NDM-1_, *bla*_VEB-3_, *bla*_SFO-1_, *bla*_TEM-1B_, *aac(3)-IId*, *mph(A)*, *dfrA27*, *ARR-3*, and two copies of *sul1* (**Figure 2A**), mediating resistance to carbapenems, β-lactams, aminoglycosides, macrolides, trimethoprim, rifampin, and sulphonamides. This plasmid could not be assigned to any known replicon type in the PlasmidFinder database (using thresholds of ≥50% identity and ≥20% coverage). However, annotation using the RAST platform suggested the presence of four putative plasmid replicons. Comparative genomic analysis revealed that KO24-256p1 shared 100% query coverage and 99.99% identity with plasmid pC39-334kb (accession no. CP061701) from *Klebsiella pneumoniae* strain C39, isolated in Zhengzhou, China, in 2019. Gene structure comparison showed that resistance genes in both plasmids were clustered in an approximately 5 kb multidrug resistance (MDR) island, flanked by multiple mobile genetic elements. In comparison, pC39-334kb harbored two additional resistance genes, *qnrA7* and an extra copy of *sul1* (**Figure 2B**). The second plasmid, KO24-256p2, was identified as an IncR-type plasmid and carried several resistance genes, including *aph(6)-Id*, *aph(3’’)-Ib*, *aadA2*, *floR*, *qnrS1*, *sul1*, *sul2*, *tet(A)*, and *dfrA12*.

### 3.4 Phylogenetic analysis

**Figure 3:**
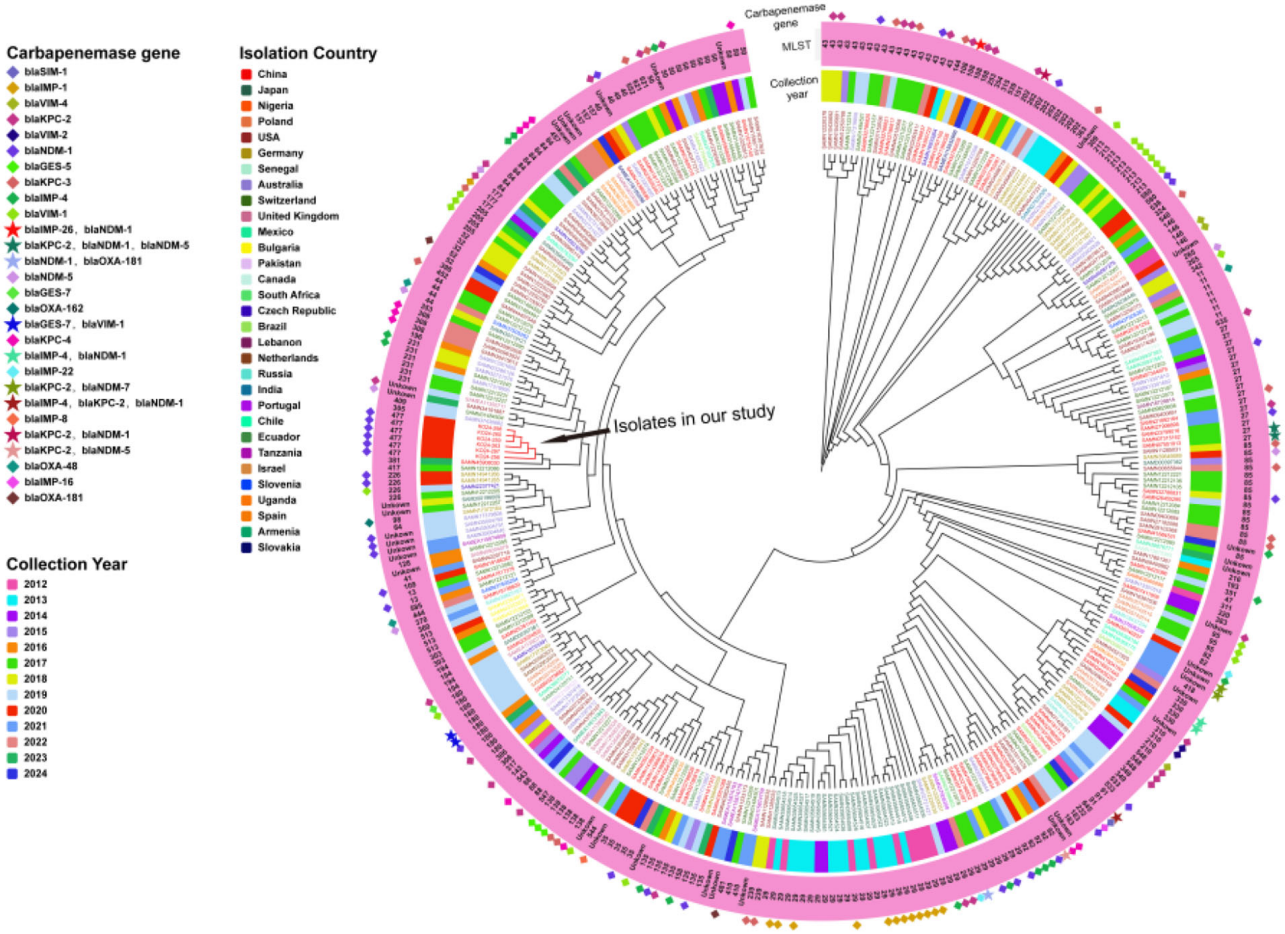
Phylogenetic tree of 344 K*. michiganensis* genomes retrieved from the PATRIC and six ST477 *K. michiganensis* clinical isolates from this study. Starting from the outermost circle, carbapenem resistance genes are represented using distinct symbols and color combinations. The second circle shows the MLST of each strain in text format. The third circle indicates the collection year of the strains using different color bars. The inner circle uses different colors to represent the countries from which the strains were isolated.

A total of 344 publicly available *K. michiganensis* genomes isolated from human hosts across the globe were retrieved from public databases and analyzed alongside the six clinical isolates from this study, resulting in a phylogenetic tree comprising 350 strains in total. These isolates originated from 31 countries, with the majority collected from the United States (n = 67), China (n = 67), Switzerland (n = 61), Japan (n = 31), Australia (n = 28), Germany (n = 25), Poland (n = 11), the United Kingdom (n = 8), the Czech Republic (n = 5), and Mexico (n = 5); the remaining 42 isolates came from other countries. The phylogenetic tree revealed a high degree of genomic homology among the isolates, with most clustering into a single evolutionary branch, indicating close relatedness. MLST typing showed genetic diversity, with the predominant sequence types being ST29 (n = 36), ST43 (n = 18), ST27 (n = 16), ST85 (n = 15), ST50 (n = 12), ST84 (n = 10), and ST180 (n = 10). Analysis of carbapenem resistance gene distribution showed that 50% (175/350) of isolates carried at least one carbapenem resistance gene, while 3.7% (13/350) harbored two or more such genes. The distribution of resistance genes varied across country: isolates from the United States predominantly carried *bla*_KPC-3_ and *bla*_KPC-4_; those from China carried *bla*_NDM-1_, *bla*_KPC-2_, and *bla*_IMP-4_; from Japan, *bla*_IMP-1_; from Australia, *bla*_NDM-1_ and *bla*_IMP-4_; from Germany, *bla*_VIM-1_; from Poland, *bla*_VIM_ variants; and from the United Kingdom, *bla*_KPC-2_ and *bla*_GES-5_. Notably, although Swiss isolates accounted for a substantial proportion (17.4%, 61/350), only two were found to carry carbapenem resistance genes. Interestingly, none of the *K. michiganensis* isolates available in public databases shared the same sequence type (ST477) as those identified in this study. The most closely related strains phylogenetically were one ST381 isolate from China and one ST417 isolate from Switzerland, neither of which harbored carbapenem resistance genes (**Figure 3**).

## 4 Discussion

Although *K. michiganensis* is less commonly encountered than *K. pneumoniae*, it has increasingly been recognized as an emerging pathogen associated with human infections (Chen et al., 2020). In 2020, an outbreak of ESBL-producing *K. michiganensis* was reported in a neonatal unit in an Australian hospital, in which contaminated detergent was suspected to be a potential source of infection (Chapman et al., 2020). In addition, in 2021, three ST92 *K. michiganensis* strains carrying *tet(X4)* and *tmexCD2-toprJ2* were isolated from pork samples in Jiangsu Province, China. Genomic comparisons revealed only a few SNP differences between these isolates and those of human origin, suggesting possible cross-host clonal transmission (Li et al., 2023a).In the present study, we report six carbapenem-resistant ST477 *K. michiganensis* strains isolated from a hospital in Jilin Province, China. Whole-genome sequencing showed that these strains differed by no more than 8 SNPs, indicating a high level of clonal relatedness. However, retrospective analysis of publicly available *K. michiganensis* genomes globally did not identify any strains with the same sequence type, suggesting that ST477 is a rare type and may represent a regionally circulating clone or a recently emerged lineage.

*K. michiganensis* is often classified within *K. oxytoca* complex in clinical settings, and large-scale studies specifically focusing on this species remain limited. In a recent multicenter study conducted by Li et al (Li et al., 2024)in 2024, 103 K*. oxytoca* complex isolates collected from 69 hospitals across China between January 2020 and November 2021 were analyzed, revealing that *K. michiganensis* accounted for a substantial proportion (46.60%, 48/103) of the isolates—a finding consistent with previous reports (Gómez et al., 2021; Wan et al., 2023). This study further demonstrated that *K. michiganensis* exhibited significantly higher *in vitro* resistance rates to commonly used antibiotics than other members of the *K. oxytoca* complex, including an 8.7% resistance rate to carbapenems. Of particular concern is a 2018 clinical isolate reported in Zhejiang Province, China, which co-harbored *bla*_KPC-2_, *bla*_NDM-1_, and *bla*_NDM-5_, and showed susceptibility only to tigecycline and colistin *in vitro* (Zheng et al., 2018). In the present study, the six *K. michiganensis* isolates harbored *bla*_NDM-1_, along with the uncommon resistance genes *bla*_VEB-3_ and *bla*_SFO-1_. Antimicrobial susceptibility testing indicated that these strains were only susceptible to amikacin, aztreonam-avibactam, eravacycline, tigecycline, and colistin. However, previous studies have reported *K. michiganensis* strains carrying the *tmexCD-toprJ* gene cluster, which confers resistance to tetracyclines via efflux mechanisms, as well as the *mcr* gene, associated with colistin resistance (Wang et al., 2021; Li et al., 2022a). These findings underscore the need for continued surveillance of emerging resistance mechanisms in this species.

Since the first discovery of NDM in India, it has rapidly disseminated worldwide. In China, the *bla*_NDM-1_ gene was first detected in *Acinetobacter baumannii* in 2011 (Chen et al., 2011). As of 15 April 2025, a total of 78 NDM variants have been recorded in the Beta-Lactamase DataBase (BLDB; http://bldb.eu/) (Naas et al., 2017). To date, *bla*_NDM_-positive *K. michiganensis* isolates have been reported in China, Japan, South Africa and other countries, where the gene is typically located on IncFIB or IncX3 plasmids (Yamada et al., 2024). In this study, we identified a *bla*_NDM-1_ gene located on a novel plasmid that harbored sequence fragments related to both IncF and IncH1 replicon types, suggesting that the plasmid may have arisen from the recombination of multiple plasmid backbones. In *K. michiganensis*, *bla*_NDM_ is commonly associated with the classical Tn*125* composite transposon structure, flanked by two copies of IS*Aba125* and frequently accompanied downstream by the *ble*_MBL_ and *trpF* genes. This configuration has been reported not only on IncX3 plasmids but also on IncFIB(K)-type plasmids (Prah et al., 2022; Jiang et al., 2023). However, in our isolate, the genetic context of *bla*_NDM-1_ differed from these classical structures: an IS*26* insertion was found upstream of a truncated IS*Aba125* element, a configuration previously observed in *Escherichia coli*, *Klebsiella pneumoniae*, *Enterobacter cloacae*, and *Citrobacter freundii* isolates (Weber et al., 2019; Zhao et al., 2021). Previous studies have demonstrated that insertion sequences (ISs) can mediate plasmid recombination and horizontal gene transfer of resistance genes, with IS*26* in particular recognized as a key driver of the rapid dissemination of antimicrobial resistance in Gram-negative bacteria (Harmer et al., 2014; He et al., 2015; Partridge et al., 2018). Our findings support this view, as multiple IS*26* elements were identified in the multidrug resistance island of KO24-256p1, potentially facilitating the integration of multiple resistance genes.

*bla*_SFO_ is a relatively rare ESBL gene that was first identified in 1999 on a plasmid from *Enterobacter cloacae* isolated in Japan (Matsumoto and Inoue, 1999). It was designated SFO-1 due to its highest homology with a β-lactamase from *Serratia fonticola*. Although *bla*_SFO-1_ remains uncommon in clinical isolates, it has been increasingly reported worldwide in recent years (Fernández et al., 2011; Chen et al., 2024; Skwor et al., 2024). Similarly, VEB (Vietnamese extended-spectrum β-lactamase) is also an infrequent ESBL that has been detected in raw meat and animals in China (A Wohlfahrtiimonas chitiniclastica with a novel type of blaVEB-1-carrying plasmid isolated from a zebra in China, 2023; Mao et al., 2024). Notably, a *bla*_VEB-3_-harboring *Enterobacter cloacae* strain was previously linked to a nosocomial outbreak in Shanghai, China (Jiang et al., 2005). Despite limited data on the genetic context of *bla*_VEB-3_, some studies suggest it is commonly associated with *intI1* (Jiang et al., 2005), while *bla*_SFO-1_ is often embedded in integron gene cassettes within transposon Tn*5393* (Zhou et al., 2017). These findings are consistent with our observations in plasmid KO24-256p1. It is likely that horizontal gene transfer and homologous recombination mediated by various mobile genetic elements contributed to the formation of the multidrug resistance island in KO24-256p1. Although most ESBLs lack the ability to hydrolyze carbapenems, resistance may still occur in the presence of porin loss or mutations, or overexpression of efflux pumps (Li et al., 2022b; Roy et al., 2022).

Through genomic analysis of *K. michiganensis* isolates derived from human hosts in public databases, we found that 50% (175/350) of the strains harbored carbapenem resistance genes, suggesting that *K. michiganensis* may serve as an important reservoir for carbapenemase genes. The global distribution of MLST types among *K. michiganensis* strains was relatively scattered, with ST29, ST43, ST27, and ST85 being the most common. Notably, ST29 strains were primarily identified in Japan, with the majority carrying the *bla*_IMP-1_ gene. ST43 and ST27 showed no clear geographic clustering and exhibited diverse carbapenem resistance gene profiles. ST85 was predominantly detected in Switzerland; however, none of these isolates carried carbapenemase genes. ST477 strains have only been identified in Jilin Province, China. Furthermore, the *bla*_NDM-1_, *bla*_VEB-3_, and *bla*_SFO-1_ resistance genes may have integrated into the *K. michiganensis* genome through mobile genetic elements. The localized clonal dissemination of these strains could be likely associated with multiple factors, including the regional prevalence of the strain, the presence of specific resistance genes, and the unique transmission dynamics in the hospital environment. Although *K. michiganensis* is less frequently isolated in clinical settings compared to *K. pneumoniae* or *E. coli*, the high prevalence of carbapenemase genes among its isolates is a concerning trend. With the rapid advancement of high-throughput sequencing technologies, the clinical relevance of *K. michiganensis* is gaining increasing attention. Our findings highlight the importance of improving the identification and surveillance of this species in clinical microbiology laboratories, particularly molecular epidemiological investigations of carbapenem-resistant strains, to enable early detection of potential outbreaks and the implementation of effective infection control strategies to mitigate its transmission risk within healthcare settings.

## 5 Conclusion

In summary, this study is the first to report the nosocomial clonal dissemination of carbapenem-resistant ST477 *K. michiganensis*. Genomic analysis revealed that resistance genes including *bla*_NDM-1_, *bla*_VEB-3_, and *bla*_SFO-1_ co-localize within a multidrug resistance region on a self-transmissible plasmid. Comparative genomics indicated that horizontal gene transfer and homologous recombination mediated by mobile genetic elements such as insertion sequences, transposons, and integrons likely play a critical role in the formation of the multidrug resistance island.

## Data Availability

The datasets presented in this study can be found in online repositories. The names of the repository/repositories and accession number(s) can be found in the article/supplementary material.
